# Supplementary material for: Relationships between Seminal Plasma Metabolites, Semen Characteristics and Sperm Kinetics in Donkey (Equus asinus)
Source: Animals (Basel). 2021 Jan 15;11(1):201. doi: 10.3390/ani11010201 (PMC7830036; doi:10.3390/ani11010201)
Supplement: Supplementary file 1 [file animals-11-00201-s001.pdf]

animals

Supplementary Material

Relationships between Seminal Plasma Metabolites, Semen Characteristics and Sperm Kinetics in Donkey (*Equus asinus*)

Maria Antonietta Castiglione Morelli [1], Angela Ostuni [1], Brunella Giangaspero [2], Stefano Cecchini [1], Augusto Carluccio [2,*] and Raffaele Boni [1,*]

[1] Department of Sciences, University of Basilicata, Campus Macchia Romana, 85100 Potenza, Italy; maria.castiglione@unibas.it (M.A.C.M.); angela.ostuni@unibas.it (A.O.); stefano.cecchini@unibas.it (S.C.)

[2] Faculty of Veterinary Medicine, University of Teramo, Loc. Piano d'Accio, 64100 Teramo, Italy; brunellagiangaspero@gmail.com

* Correspondence: acarluccio@unite.it (A.C.); raffaele.boni@unibas.it (R.B.); Tel.: +39-0861-266975 (A.C.); +39-0971-205017 (R.B.)

Citation: Castiglione Morelli, M.A.; Ostuni, A.; Giangaspero, B.; Cecchini, S.; Carluccio, A.; Boni, R. Relationships between Seminal Plasma Metabolites, Semen Characteristics and Sperm Kinetics in Donkey (*Equus asinus*). *Animals* **2021**, *11*, 201. https://doi.org/10.3390/ani11010201

Received: 16 November 2020
Accepted: 12 January 2021
Published: 15 January 2021

Publisher's Note: MDPI stays neutral with regard to jurisdictional claims in published maps and institutional affiliations.

Copyright: © 2021 by the authors. Licensee MDPI, Basel, Switzerland. This article is an open access article distributed under the terms and conditions of the Creative Commons Attribution (CC BY) license (http://creativecommons.org/licenses/by/4.0/).

Table 1. Correlation coefficients (R) between donkey seminal plasma metabolites evaluated by ^{1}H NMR spectroscopy (extended version).

	Alanine	Benzoate	Carnitine	Choline	Citrate	Creatine	Glutamate	Glycerol	GPC	Hippurate	Lactate	Leucine	myo-inositol	O-Acetyl carnitine	Phenyl alanine	Trimethylamine N-oxide
Acetate	+0.514	+0.171	+0.396	+0.557	+0.745 **	+0.469	+0.596 *	+0.416	+0.571	−0.377	+0.486	+0.414	+0.335	+0.559	+0.191	+0.083
Alanine		−0.020	+0.923 **	+0.784 **	+0.544	+0.969 **	+0.938 **	+0.164	+0.751 **	−0.141	−0.041	+0.852 **	+0.696 *	+0.754 **	+0.015	+0.709 **
Benzoate			+0.204	+0.190	+0.541	−0.048	−0.014	−0.126	−0.046	+0.583	+0.441	+0.278	+0.067	−0.028	+0.440	−0.053
Carnitine				+0.776 **	+0.622 *	+0.873 **	+0.874 **	+0.008	+0.763 **	−0.009	+0.068	+0.907 **	+0.658 *	+0.606 *	+0.267	+0.719 **
Choline					+0.697 *	+0.781 **	+0.767 **	−0.091	+0.650 *	−0.021	+0.295	+0.652 *	+0.902 **	+0.852 **	+0.092	+0.465
Citrate						+0.541	+0.600 **	+0.178	+0.505	−0.061	+0.690 *	+0.707 **	+0.513	+0.450	+0.218	+0.345
Creatine							+0.908 **	+0.055	+0.666 *	−0.053	−0.031	+0.847 **	+0.781 **	+0.759 **	+0.019	+0.776 **
Glutamate								+0.222	+0.890**	−0.106	−0.076	+0.769 **	+0.625 *	+0.755 **	−0.204	+0.527
Glycerol									+0.153	−0.517	+0.054	+0.125	−0.290	+0.009	+0.487	−0.137
GPC										−0.201	−0.101	+0.562	+0.421	+0.580	−0.235	+0.276
Hippurate											−0.278	−0.039	+0.108	+0.016	+0.102	−0.080
Lactate												+0.242	+0.196	+0.011	+0.496	+0.095
Leucine													+0.614 *	+0.416	+0.364	+0.845 **
Myo-inositol														+0.746 **	+0.166	+0.623 *
O-Acetyl carnitine															−0.259	+0.262
Phenyl alanine																+0.487

* ($p < 0.05$), ** ($p < 0.01$).

Table 2. Correlation coefficients (R) between seminal plasma metabolites and sperm kinetics evaluated in the ten donkey stallions at either time of semen collection (T0) or after 24h storage at 4°C (T24). TotMot (total sperm motility), Prog (progressive sperm motility), VCL (curvilinear velocity), VSL (straight-line velocity) and VAP (average path velocity). Extended version.

	**T0**					**T24**				
	**Tot-Mot**	**Prog**	**VCL**	**VSL**	**VAP**	**Tot-Mot**	**Prog**	**VCL**	**VSL**	**VAP**
Acetate	–0.340	–0.744 *	–0.045	–0.593	–0.067	–0.434	–0.508	–0.144	–0.161	–0.098
Alanine	+0.142	–0.343	–0.136	–0.074	+0.064	+0.086	–0.049	+0.082	+0.118	+0.171
Benzoate	–0.029	–0.095	+0.556	+0.441	+0.689 *	+0.392	+0.323	+0.645 *	+0.473	+0.534
Carnitine	+0.242	–0.257	–0.029	+0.121	+0.248	+0.353	+0.206	–0.398	+0.391	+0.449
Choline	–0.474	–0.606	–0.525	–0.374	–0.255	–0.076	–0.087	+0.147	+0.152	+0.201
Citrate	–0.341	–0.760 **	+0.278	–0.378	+0.221	+0.023	–0.032	+0.386	+0.304	+0.380
Creatine	+0.101	–0.384	–0.103	–0.099	+0.035	+0.061	–0.021	+0.059	+0.104	+0.152
Glutamate	–0.067	–0.568	–0.124	–0.318	–0.131	–0.099	–0.245	–0.032	–0.057	–0.003
Glycerol	+0.015	–0.389	+0.176	–0.306	+0.016	–0.350	–0.479	–0.331	–0.352	–0.336
Glyceropho-sphocholine	–0.279	–0.564	–0.312	–0.408	–0.290	–0.177	–0.354	–0.084	–0.142	–0.079
Hippurate	+0.101	+0.173	+0.360	+0.540	+0.362	+0.292	+0.304	+0.292	+0.149	+0.160
Lactate	–0.288	–0.286	+0.136	-0.333	+0.099	+0.093	+0.188	+0.466	0.474	+0.474
Leucine	+0.216	–0.339	+0.268	+0.174	+0.465	+0.402	+0.280	+0.450	+0.448	+0.511
Myo-inositol	–0.408	–0.439	–0.522	–0.182	–0.234	–0.016	+0.056	+0.109	+0.156	+0.186
O-Acetyl carnitine	–0.135	–0.374	–0.333	–0.366	–0.284	–0.292	–0.308	–0.158	–0.129	–0.102
Phenylalanine	+0.326	+0.517	+0.226	+0.565	+0.558	+0.821 **	+0.853 **	+0.847 **	+0.902 **	+0.909 **
Trimethylamine N-oxide	+0.316	+0.036	+0.033	+0.266	+0.271	+0.487	+0.466	+0.371	+0.474	+0.497
pH	–0.640	–0.560	–0.238	–0.232	+0.078	–0.554	–0.487	–0.446	–0.434	–0.412
Osmolarity	–0.666 *	–0.532	–0.120	–0.025	+0.106	–0.249	–0.273	–0.058	–0.184	–0.141
Semen volume	+0.082	–0.136	+0.549	+0.470	+0.736 *	+0.425	+0.375	+0.591 *	+0.484	+0.555
Sperm concentration	+0.017	–0.064	–0.491	–0.116	–0.421	+0.324	+0.256	+0.360	+0.357	+0.387
Sperm production	+0.156	–0.181	+0.339	+0.394	+0.507	+0.121	+0.055	+0.170	+0.203	+0.249
TotMot		+0.742 *	+0.599	+0.476	+0.457		+0.951 **	+0.757 **	+0.900 **	+0.892 **
Prog			+0.183	+0.507	+0.156			+0.867 **	+0.916 **	+0.886 **
VCL				+0.332	+0.668 *				+0.966 **	+0.975 **
VSL					+0.783 **					+0.994 **

* ($p < 0.05$), ** ($p < 0.01$).
